# Transient Otoacustic Emissions with tone pip in Individuals with Sensorineural Hearing Loss

**DOI:** 10.1590/S1808-86942011000500014

**Published:** 2015-10-22

**Authors:** Thays Bueno Takeda, Daniela Gil

**Affiliations:** 1 Specialist, speech therapist; 2 Doctoral degree. Adjunct professor in the Hearing Disorders Discipline, Sao Paulo Federal University (Universidade Federal de São Paulo)

**Keywords:** hearing, hearing loss, sensorineural, speech, language and hearing sciences

## Abstract

**Abstract:**

Otoacoustic Emissions are generated by the cochlea in response to sound stimuli. They can be generated by clicks or specific frequency stimuli, such as tone pips. This is a quick and objective test with several applications.

**Objective:**

To investigate the influence of the type of stimulus achieving otoacoustic emissions in individuals with mild and moderate sensorineural hearing loss of sloping configuration.

**Material and Method:**

Thirty-two male and female patients aged from 17 to 63 years, with symmetric sensorineural hearing loss with a sloping configuration were evaluated. All subjects underwent transient otoacoustic emissions testing elicited by clicks and 2.000Hz and 4.000Hz tone pips.

**Results:**

The degree of hearing loss and gender influenced otoacoustic emissions; it was significant for click stimulus and tone pips at 2.000Hz. Emissions were absent more often in females with both procedures.

**Conclusions:**

Otoacoustic emissions evoked with clicks coincided with the emissions of tone pips at 2.000Hz. Tone pips at 4.000Hz were more sensitive than clicks for detecting impairment in individuals with high frequencies hearing loss. Gender and the degree of hearing loss ere factors that affected OAE registration.

## INTRODUCTION

Otoacoustic emissions (OAEs) consist of low intensity sound energy that after amplification may be recorded in the outer ear canal. OAEs originate in the cochlea, specifically in outer hair cells^1^,^2^.

OAEs are an important tool for objectively evaluating the function of the inner ear, especially the outer hair cells of the cochlea^1^,^2^.

It is a quick, objective, non-invasive, and easily applied procedure that may be done at any age^3^.

OAEs are not used for quantifying hearing loss, but rather to detect their presence, as they are present in all normally functioning ears. OAEs become absent when behavioral auditory thresholds are above 30 dBNA, that is, when there is any degree - even if mild - of hearing loss^2^,^4^,^5^.

OAEs have several clinical uses, including neonatal and school-age hearing screening, in the differential diagnosis of cochlear and retrocochlear hearing losses, in monitoring subjects exposed to noise or ototoxic drugs, in analyzing and defining the progression of sudden and progressive hearing loss, and in studying cochlear dysfunction. OAEs may be tested in all age groups, including those that are hard to test^3^.

Transient otoacoustic emissions (TOAEs) are responses evoked by short stimuli, such as clicks at 500 to 6,000 Hz. OAE responses may be seen per frequency band. However, these responses are due to a filtering process in the equipment, which separates them by frequency band. Click-generated responses do not necessarily match the cochlear region involved in that specific frequency; they reflect a global cochlear response^6^.

A more specific method for evaluating OAEs by frequency is to use a tone pip stimulus. Tone pips present the energy as a single frequency pure tone uncontaminated by the frequencies of adjacent energies. This stimulus causes the basilar membrane of the cochlea to move in the frequency cochlear region that coincides with the stimulus frequency^6^.

Low frequencies are preserved - or at least with lower auditory thresholds - in sloping sensorineural hearing loss. Patients with this type of hearing loss may present TOAEs when stimulated by clicks because of lack of frequency selectivity in responses.

The purpose of this study was to compare TOAEs with clicks and tone pips at 2,000 Hz and 4,000 Hz in subjects with mild and moderate sloping sensorineural hearing loss; the variables were gender, degree of hearing loss, and first ear to be tested.

## MATERIAL AND METHODS

The institutional review board analyzed and approved the study design (no. 01988/08). All subjects were informed beforehand about the procedures, and participants signed a free informed consent form.

Inclusion criteria were: being a patient of the institution, having mild or moderate bilateral sloping sensorineural hearing loss, presenting a bilateral type A tympanometric curve, and aged from 15 to 65 years.

A clinical history was taken of the selected patients, followed by visual inspection of the outer ear canal, pure tone and voice audiometry, acoustic immittance testing, and recording of TOAEs with clicks and tone pips.

OAEs were recorded by placing a probe in the outer ear canal - this probe has a miniature microphone that seals the outer ear canal with an olive-shaped tip. The stimulus passes from an amplifier to an analog-digital converter and a digital signal processor, which makes it possible to analyze the recorded sounds spectrally and to separate them from other sounds generated in the body.

TOAEs were recorded following a click and then a tone pip at 2,000 Hz and 4,000 Hz. Testing took place in a non-acoustically lined silent room; the testing device was an ILO 88 - otoacoustic emissions analyzer (Otodynamics Ltd., version 88) coupled to a microcomputer. The probe was placed only once; OAEs were recorded with both stimuli first on one side and then on the other. Testing started on the right ear in half of the sample, and on the left ear in the other half.

A 260 click recording was used; the gain was adjusted to keep stimulus intensities between 75 and 85 dB in the full-menu mode (20 ms window). The criteria for the presence of responses was a general reproducibility correlation of at least 50%, by tested frequency (3 dB above signal to noise - S/R), and general amplitude (response), and wave stability above 70% with both stimuli.^3^

OAEs with tone pips required the equipment to be modified. Before testing, the examiner selected on the screen: “*menu”*, “*stimulus”*, “*tone pip”* and entered the frequency at which testing was done - first 2,000 Hz, then 4,000 Hz.

Statistical analysis of the data aimed at comparing OAEs obtained with clicks and tone pips at 2,000 Hz and 4,000 Hz. The equality of two proportions test was applied, as well as measurements of the median, standard deviation, 1^st^ and 3^rd^ quartiles, and confidence intervals at a 95% statistical confidence level.

Results where statistical significance was attained are marked with the symbol (*); the p-value was 0.05.

## RESULTS

The results of comparing TOAEs evoked by clicks and tone pips at 2,000 Hz and 4,000 Hz are presented below.

Table 1 shows the mean values of OAEs for the response, reproducibility, stability and stimulus in all tested ears.

**Table 1:** Descriptive measures for the parameters response, reproducibility, stability, and OAEs with clicks and tone pips at 2,000 Hz and 4,000 Hz, for both ears and different levels of hearing loss.

Descriptive		Mean	Median	Standard Deviation	Q1	Q3	N	CI
	Click	10.70	9.2	4.22	7.0	14.3	22	1.76
Resp.	Tone 2k	7.38	6.5	2.82	5.4	8.4	22	1.18
	Tone 4k	6.49	5.7	2.47	5.0	7.1	16	1.21
	Click	13.6%	7.5%	43.9%	-24.5%	59.3%	64	10.7%
Repro.	Tone 2k	4.0%	5.0%	30.4%	-21.5%	24.0%	64	7.5%
	Tone 4k	-6.6%	-12.0%	30.7%	-28.3%	17.0%	64	7.5%
	Click	86.2%	88.0%	9.0%	76.8%	94.0%	64	2.2%
Stab.	Tone 2k	82.9%	85.0%	12.1%	79.0%	89.0%	64	3.0%
	Tone 4k	85.0%	84.0%	6.3%	80.0%	91.0%	64	1.6%
	Click	78.05	78	2.31	76	79	64	0.56
Stim.	Tone 2k	78.98	79	2.49	77.75	80	64	0.61
	Tone 4k	80.52	81	2.52	79	82	64	0.62

Key: Resp: Response

Repro: Reproducibility

Stab: Stability

Stim: Stimulus

Q1: 1^st^ quartile

Q3: 3^rd^ quartile

N: Number of total OAEs

CI: Confidence interval

The response amplitude ranged from 4.3 dB to 17.8 dB (mean - 8.19 dB), and was higher with clicks, followed by tone pips at 2,000 Hz and by tone pips at 4,000 Hz. Similarly, reproducibility was higher with OAEs evoked by click, followed by tone pips at 2,000 Hz and tone pips at 4,000 Hz; it ranged from - 67% to 84% (mean - 3.6%). Stability was higher than 70% (mean - 84.7%), and the stimulus level was between 75 dB and 85 dB (mean - 79.18 dB) for three stimuli according to click parameters defined in the literature.

Table 2 shows the distribution of OAEs evoked by clicks and tone pips at 2,000 Hz and 4,000 Hz according to the criterion present/absent per ear.

**Table 2:** Result of OAEs evoked by clicks and tone pips at 2,000 Hz and 4,000 Hz, in both ears and according to the degrees of hearing loss.

		Click		Tone Pip 2kHz		Tone Pip 4kHz	
		N	%	N	%	N	%
	RE	11	34.4%	11	34.4%	3	9.4%
Present	LE	11	34.4%	11	34.4%	4	12.5%
	RE	21	65.6%	21	65.6%	29	90.6%
Absent	LE	21	65.6%	21	65.6%	28	87.5%

Key: RE: right ear. LE: left ear.

This Table shows that OAEs evoked by clicks and tone pips at 2,000 Hz were similar. There was a high rate of absent responses for OAEs evoked by tone pips at 4,000 Hz; this was compatible with the type of hearing loss that was studied - auditory thresholds at high frequencies were above 25 dB, where OAEs are not expected.

Table 3 shows the *p*-values of Table 2.

**Table 3:** *P*-values of Table 2.

		Click
	tone pip 2kHz	1.000
RE	tone pip 4kHz	0.016*
	tone pip 2kHz	1.000
LE	tone pip 4kHz	0.039*

Significance: *p* < 0.05

Key: RE: right ear. LE: left ear.

Table 3 shows that there was a statistically significant difference in OAEs evoked by click and tone pip at 4,000 Hz in both ears.

Table 4 presents a comparison of OAEs evoked by clicks and tone pips at 2,000 Hz and 4,000 Hz according to the variable gender.

**Table 4:** Results of OAEs evoked by clicks and tone pips at 2,000 Hz and 4,000 Hz, according to sex.

		Female		Male		
Gender		N	%	N	%	p-value
	Absent	20	100.0%	22	50.0%	
Click	Present	0	0.0%	22	50.0%	<0.001*
	Absent	18	90.0%	24	54.5%	
Tone 2k	Present	2	10.0%	20	45.5%	0.006*
	Absent	17	85.0%	40	90.9%	
Tone 4k	Present	3	15.0%	4	9.1%	0.483

Significance *p* < 0.05.

The variable gender affected the recording of OAEs; it was statistically significant for OAEs evoked using clicks and tone pips at 2,000 Hz, with more absent results in women for clicks and tone pips at 2,000 Hz. There were more absences than presences of OAEs with tone pips at 4,000 Hz in both sexes, which was not statistically significant.

Table 5 compares OAEs evoked using clicks and tone pips at 2,000 Hz and 4,000 Hz according to the degree of hearing loss.

**Table 5:** Results of OAEs evoked by clicks and tone pips at 2,000 Hz and 4,000 Hz according to the degree of hearing loss.

		Mild		Moderate		
Loss		N	%	N	%	*p*-value
	Absent	0	0.0%	42	91.3%	
Click	Present	18	100.0%	4	8.7%	<0.001*
	Absent	0	0.0%	42	91.3%	
Tone 2k	Present	18	100.0%	4	8.7%	<0.001*
	Absent	16	88.9%	41	89.1%	
Tone 4k	Present	2	11.1%	5	10.9%	0.978

Significance *p* < 0.05

The variable degree of hearing loss affected the recording of OAEs; it was statistically significant for clicks and tone pips at 2,000 Hz, where there was complete agreement between both stimuli types. In both cases, emissions were present in subjects with mild hearing loss, and absent in subjects with moderate hearing loss. The comparison between OAEs evoked by clicks ant tone pips at 4,000 Hz showed that emissions were absent in most cases of moderate hearing loss.

## DISCUSSION

This study showed that the amplitude of TOAEs evoked by specific frequency stimuli (tone pips) decreased as the stimulus frequency increased, which concurred with another published study^7^. Table 1 shows this result, where the response amplitude was lower when the tone pip in the test was 4,000 Hz, followed by 2,000 Hz tone pips, and clicks. The mean response generated by tone pips at 4,000 Hz was 6.49 dB, while for tone pips at 2,000 Hz it was 7.38 dB; the response generated by clicks was 10.70 dB - these differences were not statistically significant.

Clicks of any intensity for evoking OAEs are powerful activators of efferent activity, while tone bursts and/ or tone pips activate this system less effectively, as they stimulate fewer efferent fibers^8^. Table 1 shows published results, where the general response amplitude of OAEs in this study ranged from 4.3 dB to 17.8 dB (mean - 8.19 dB), and was higher when evoked by clicks, followed by tone pips at 2,000 Hz and tone pips at 4,000 Hz.

As in our study, McPherson et al. (2006) included tone burst-evoked OAEs in their protocol to reduce falsepositive results in neonatal screening. These authors found, however, that tone burst responses at 1,000 Hz, 2,000 Hz, and 3,000 Hz were consistently higher and more effective compared with clicks^9^. Our results differed in that the click-evoked response amplitude was 10.70 dB, which was higher than tone pip responses at 2,000 dB and 4,000 Hz (respectively 7.38 dB and 6.49 dB) (Table 1).

According to some authors, tympanometric measures and the intensity of the recorded stimulus in the outer ear canal affected the response level of TOAEs (significant positive correlation)^10^. In the present study, a type A tympanometric curve and parameters for stimulus intensity from 75 dB to 85 dB were inclusion criteria; as seen in Table 1, the stimulus level ranged from 78.05 dB to 80.52 dB (mean - 79.18 dB). Reproducibility and stability were also conditions for recording and interpreting TOAEs. Reproducibility was observed in a higher percentage of OAEs when using clicks, followed by tone pips at 2,000 Hz and at 4,000 Hz, ranging from 67% to 84% (mean - 3.6%). Stability was above 70% (mean - 84.7%). Also in the present study, parameters for analyzing responses, reproducibility, stability, and stimulus were those considered as ideal in the literature; the same applied to parameters for recording and analyzing the presence or absence of OAEs^3^.

A few authors have found consistent responses for emissions using tone bursts at 1,000 Hz, 2,000 Hz and 4,000 Hz in normal-hearing and hearing-impaired children. These authors found that tone bursts at 2,000 Hz and 4,000 Hz discriminated normal and altered ears more effectively, and concluded that tone bursts were superior to clicks for identifying hearing loss^11^. Likewise, Table 2 shows the same relation between different frequencies, where OAEs evoked with clicks and tone pips at 2,000 Hz and 4,000 Hz were separated by the presence of absence of these stimuli and the ear side. We found that OAEs with clicks and tone pips at 2,000 Hz had similar results, demonstrating an agreement between stimulus types. OAEs evoked by tone pips at 4,000 Hz, on the other hand, had a high rate of absent responses, which was compatible with the type of hearing loss in which auditory thresholds at high frequencies were above 25 dB, and no OAE responses were expected.

Table 3 shows that there was a statistically significant difference in a comparison of OAEs with clicks and tone pips at 4,000 Hz in both ears. This difference suggested that tone pips were more accurate than clicks for identifying high frequency hearing loss in subjects with mild and moderate sloping sensorineural hearing loss, demonstrating an important clinical use for this type of stimulus.

Our findings differ from those of a few authors that recorded TOAEs in young adults^10^. Gender was one of the factors that affected the response level of OAEs; the response amplitude of emissions was higher in females than males; this again differed from the findings presented in Table 4, which shows that males had higher amplitude responses than females. It should be made clear, however, that the sample was not sex-paired; there were more female subjects with moderate than mild hearing loss, which may explain these differences.

A study of OAEs evoked by tone bursts showed advantages for the right ear, female gender, and neonates with no risk indicators for hearing loss, but which was not statistically significant^12^; this again differed from our results, in which males had superior responses. Table 4 shows OAEs evoked by clicks and tone pips at 2,000 Hz and 4,000 Hz separated by gender. The variable gender affected the recording of OAEs, and was statistically significant for tests done with clicks and tone pips at 2,000 Hz; there was a higher rate of absence of OAEs in females in both stimuli. It should be said that there were more female subjects with moderate hearing loss, which may have led to this results; furthermore, the sample population consisted of adults, rather than neonates.

The hearing level was also a significant factor in some published papers and in the present study; subjects with more severe hearing loss did not respond (Table 5). We found a 100% presence of responses for OAEs evoked by clicks and tone pips at 2,000 Hz in subjects with mild hearing loss but with normal hearing at the tested frequency. Absence of OAEs was more evident (88.9%) when evoked by tone pips at 4,000 Hz in mild hearing loss, and complete for all subjects with moderate hearing loss, all of which had auditory thresholds worse than 25 dB at the tested frequency.

Our findings concur with those of other authors that tested OAEs in normal-hearing subjects and in patients with sensorineural hearing loss. These authors found that OAEs were present in normal-hearing individuals, and absent in subjects with auditory thresholds worse than 35 dB^4^. Similarly, we found that OAE responses were present in 100% of normal-hearing subjects at the tested frequency (Table 5). OAEs were absent in all subjects with hearing loss at the tested frequency (4,000 Hz).

The present study is very important for clinical practice, as it suggests that recording OAEs with clicks combined with tone pips add to the hearing loss detection protocols. This may reduce the need for second visits for retesting, and decrease false-positives, especially in subjects with mild and moderate sloping sensorineural hearing loss.

## CONCLUSION

Based on an assessment of subjects with mild and moderate sloping sensorineural hearing loss using TOAEs evoked by clicks and tone pips at 2,000 Hz and 4,000 Hz, we concluded that:

• Click-evoked OAEs coincide with OAEs evoked by tone pips at 2,000Hz.
• The variable degree of hearing loss affected the recording of OAEs, and was statistically significant for recordings with click and 2,000 Hz tone pip stimuli.
• Gender affects the recording of OAEs, and was statistically significant for recordings with click and 2,000 Hz tone pip stimuli; there were more absence of OAEs in female subjects in both procedures.
